# Sonographic Pearls for Imaging the Brachial Plexus and Its Pathologies

**DOI:** 10.3390/diagnostics10050324

**Published:** 2020-05-20

**Authors:** Po-Cheng Hsu, Ke-Vin Chang, Kamal Mezian, Ondřej Naňka, Wei-Ting Wu, Yi-Chiang Yang, Stefan Meng, Vincenzo Ricci, Levent Özçakar

**Affiliations:** 1 Department of Physical Medicine and Rehabilitation, National Taiwan University Hospital, Bei-Hu Branch, Taipei 10845, Taiwan; myronrbman@gmail.com (P.-C.H.); wwtaustin@yahoo.com.tw (W.-T.W.); 2 Department of Physical Medicine and Rehabilitation, National Taiwan University College of Medicine, Taipei 10048, Taiwan; 3 Department of Rehabilitation Medicine, Charles University, First Faculty of Medicine and General University Hospital in Prague, 12800 Prague, Czech Republic; kamal.mezian@gmail.com; 4 Institute of Anatomy, Charles University, First Faculty of Medicine, 12800 Prague, Czech Republic; ondrej.nanka@lf1.cuni.cz; 5 Department of Physical Medicine and Rehabilitation, Taipei Veterans General Hospital, Taipei 11221, Taiwan; yichiang2312@gmail.com; 6 Center for Anatomy and Cell Biology, Medical University of Vienna and Radiology, Hanusch Hospital, 1090 Vienna, Austria; stefan.meng@muv.ac.at; 7 Department of Biomedical and Neuromotor Science, Physical and Rehabilitation Medicine Unit, Istituto di Ricovero e Cura a Carattere Scientifico Rizzoli Orthopedic Institute, 40136 Bologna, Italy; vincenzo.ricci58@gmail.com; 8 Department of Physical and Rehabilitation Medicine, Hacettepe University Medical School, 06100 Ankara, Turkey; lozcakar@yahoo.com

**Keywords:** sonography, neck, brachial plexus, nerve, injury

## Abstract

The brachial plexus (BP) is a complicated neural network, which may be affected by trauma, irradiation, neoplasm, infection, and autoimmune inflammatory diseases. Magnetic Resonance Imaging is the preferred diagnostic modality; however, it has the limitations of high cost and lack of portability. High-resolution ultrasound has recently emerged as an unparalleled diagnostic tool for diagnosing postganglionic lesions of the BP. Existing literature describes the technical skills needed for prompt ultrasound imaging and guided injections for the BP. However, it remains particularly challenging for beginners to navigate easily while scanning its different parts. To address this, we share several “clinical pearls” for the sonographic examination of the BP as well as its common pathologies.

## 1. Introduction

The brachial plexus (BP) is considered the most complicated area of the peripheral neural network traversing from the cervical intervertebral foramina to the axilla. It can be affected by trauma, irradiation, neoplasm, infection, and autoimmune inflammatory diseases [1]. Magnetic resonance imaging (MRI) is the preferred diagnostic modality for preganglionic and postganglionic lesions [1,2]. Recently, high-resolution ultrasound (US) has emerged as the most powerful tool to assess peripheral nerve disorders [3,4]. Although it is limited to the detection of preganglionic lesions (due to overlying bony components), US has advantages of portability, cost-effectiveness, real-time imaging, and ease of access for guided interventions over MRI. There are several well-written protocols explaining technical skills needed for US imaging and guided interventions of the BP [5,6,7]. However, it remains challenging, particularly for beginners, to navigate promptly while evaluating different parts and pathologies of the BP. To address this, we share several “clinical pearls” with a focus on the sonographic examination of the BP and its relevant pathologies.

## 2. Anatomy

**Figure 1:**
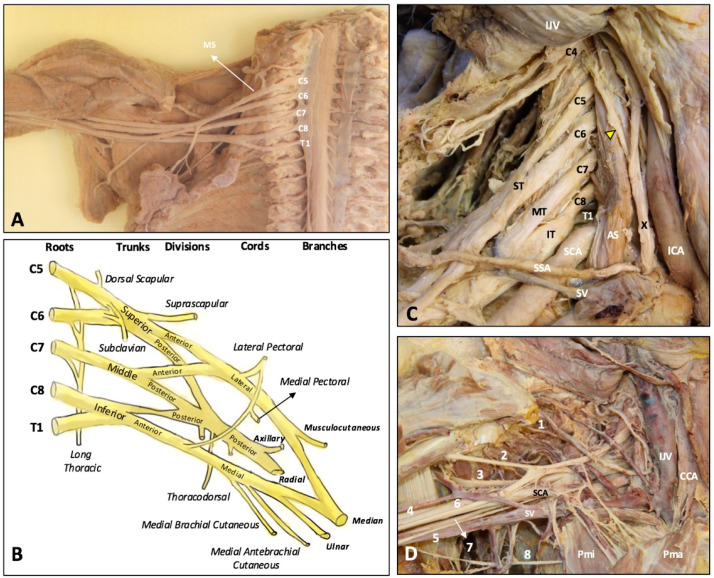
Overview of the brachial plexus on the cadaveric model after removal of the anterior scalene muscle (**A**). Schematic drawing of the brachial plexus anatomy (**B**). The roots, trunks, divisions, cords (**C**), and terminal branches (**D**) of the brachial plexus in the cadaveric models. 1: suprascapular nerve; 2: musculocutaneous nerve; 3: axillary nerve; 4: radial nerve; 5: medial brachial cutaneous nerve; 6: median nerve; 7: ulnar nerve; 8: intercostobrachial cutaneous nerve. AS: anterior scalene muscle; MS: middle scalene muscle; CCA: common carotid artery; ICA: internal carotid artery; IJV: internal jugular vein; SCA: subclavian artery; SV: subclavian vein; SSA: suprascapular artery; ST: superior trunk; MT: middle trunk; IT: inferior trunk; Pma: pectoralis major muscle; Pmi: pectoralis minor muscle; X: vagus nerve. Yellow arrowhead: phrenic nerve.

The upper extremity is innervated by the C5 to T1 anterior rami, often referred to as the roots, of the brachial plexus (Figure 1A) which has three trunks, six divisions, three cords and five major peripheral nerves (Figure 1B–D) [5]. The C5 and C6 anterior rami constitute the superior trunk, C8 and T1 anterior rami constitute the inferior trunk, and C7 solely forms the middle trunk. All the three trunks give off an anterior and a posterior division. The three posterior divisions merge to form the posterior cord. Whereas the anterior divisions of the superior and middle trunks give rise to the lateral cord, and the anterior division of the inferior trunk forms the medial cord [5]. The lateral, medial, and posterior cords are named based on their reciprocal position to the subclavian artery [8]. The posterior cord gives off the radial and axillary nerves, the lateral cord gives off the musculocutaneous nerve, and the medial cord gives off the ulnar nerve. The median nerve is composed of both lateral and medial cords [9].

## 3. Imaging for Normal BP

All the US images in this manuscript were obtained using a 5–18-MHz linear transducer (HI VISION Ascendus, Hitachi, Japan).

### 3.1. Should We Start the Scanning Cranially?

**Figure 2:**
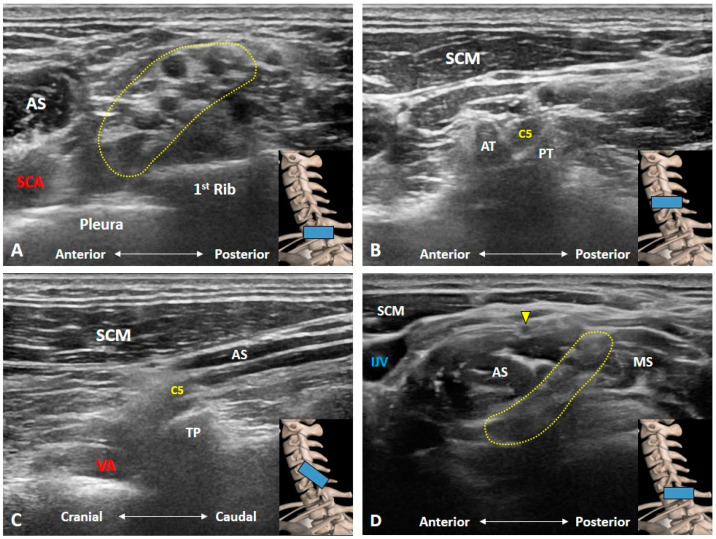
Ultrasound imaging of the brachial plexus. It resembles a bunch of grapes, situated posterior to the subclavian artery (**A**). The C5 anterior ramus is seen in its short-axis (**B**) and long-axis (**C**) views. In some variants, the C5 anterior ramus (yellow arrowhead) may course anterior to the anterior scalene muscle instead of the inter-scalene groove (**D**). AS: anterior scalene muscle; AT: anterior tubercle; IJV: internal jugular vein; MS: middle scalene muscle; PT: posterior tubercle; SCA: subclavian artery; SCM: sternocleidomastoid muscle; TP: transverse process; VA: vertebral artery. The yellow dashed area encircles the brachial plexus and the blue rectangles at the bottom-right corners represent the transducer position.

Scanning of the BP is highly recommended to be initiated from the supraclavicular fossa [7]. This enhances the chances of recognition of BP, resembling a bunch of grapes, situated posterior to the subclavian artery (Figure 2A). Traditionally, physicians were advised to start scanning from the cranial part of the BP, e.g., C5 anterior ramus, after identifying its corresponding transverse processes in a horizontal plane (Figure 2B). While the transverse processes project to the horizontal plane, anterior rami course inferiorly and laterally as they leave the inter-tubercular grooves. The angle between the transverse processes and the extraforaminal anterior rami impedes clear visualization of both structures (Figure 2C). Therefore, novice users may have difficulty in identifying and visualizing the cranial part of the BP, as they will be focusing on distinguishing different bony configurations of the transverse process. Visualization of BP distally and tracing it proximally will avoid this difficulty altogether. In case of variations, cervical anterior rami may course anterior to the anterior scalene muscle or pierce the muscle belly instead of traveling inside the inter-scalene groove [10]. As such, extra attention is a prerequisite when using the caudal-to-cranial tracking technique (Figure 2D and Supplementary Video S1).

### 3.2. Is the C7 Transverse Process Easily Recognized?

**Figure 3:**
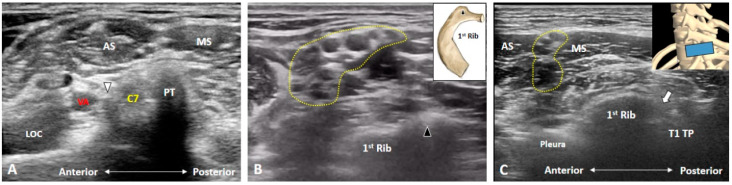
The C7 transverse process usually has only one posterior tubercle. In certain cases, a rudimentary anterior tubercle (white arrowhead) can be visualized (**A**). The costal tubercle (black arrowhead) on the first rib may be mistaken as the posterior tubercle of the transverse process (**B**). Swapping the transducer posteriorly to validate the first costotransverse joint (white arrow) helps differentiating the first rib from the C7 transverse process (**C**). AS: anterior scalene muscle; LOC: longus colli muscle; MS: middle scalene muscle; PT: posterior tubercle; TP: transverse process; VA: vertebral artery. The yellow dashed area encircles the brachial plexus and the blue rectangle represents the transducer position.

**Figure 4:**
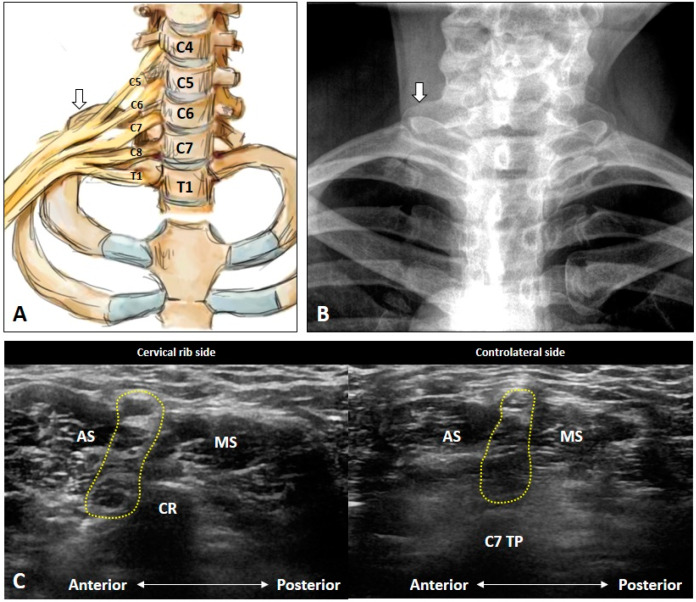
Schematic drawing (**A**), X-ray imaging (**B**) and ultrasound images (**C**) of the cervical rib (white arrows). AS: anterior scalene muscle; CR: cervical rib; MS: middle scalene muscle; TP: transverse process. The yellow dashed area encircles the brachial plexus.

Unlike the transverse processes of the C3 to C6 vertebrae, the C7 transverse process usually has only one posterior tubercle which is commonly used as a sonographic landmark to locate the C7 anterior ramus [5]. However, in certain cases, a rudimentary anterior tubercle might be visualized at the C7 transverse process (Figure 3A). Therefore, following the above stated caudal-to-cranial nerve tracking technique can effectively prevent misrecognition of the cervical anterior ramus level in such cases. However, the costal tubercle on the first rib may occasionally be mistaken as the posterior tubercle of the C7 transverse process (Figure 3B); swapping the transducer posteriorly and validating the first costotransverse joint (Figure 3C) help in differentiating the two. Alternately, one can confirm the attachment from the anterior and middle scalene muscles. Furthermore, if an echogenic shadow is visualized at the C7 level, a cervical rib should be highly suspected and swapping the transducer back and forth will clarify its connection with the C7 transverse process (Figure 4).

### 3.3. How Can We Locate the C8 and T1 Roots?

**Figure 5:**
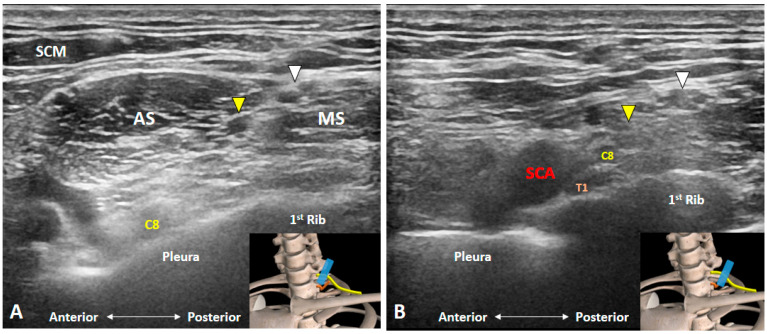
Ultrasound imaging of the C8 and T1 anterior rami. The C8 anterior ramus (**A**) is seen on top of the pleura at the bottom of the inter-scalene groove. T1 anterior ramus (**B**) can be visualized distal and lateral to the inter-scalene groove, emerging from the undersurface of the first rib. AS: anterior scalene muscle; MS: middle scalene muscle; SCA: subclavian artery; SCM: sternocleidomastoid muscle. White arrowheads: superior trunk; Yellow arrowheads: middle trunk. The blue rectangles at the bottom-right corners represent the transducer position.

The C8 anterior ramus emerges from the intervertebral foramen of C7/T1 and courses caudal to the C7 transverse process [5]. It can be easily depicted at the inter-scalene groove wherein the C5 to C8 anterior rami are arranged in a line between the anterior and middle scalene muscles with the C5 anterior ramus being most superficial and the C8 being the deepest (Figure 5A and Supplementary Video S2). Moreover, the C8 anterior ramus is cranial to the T1 transverse process coursing on top of the first rib. The T1 anterior ramus arises from the intervertebral foramen of T1/T2 and can be visualized distal and lateral to the inter-scalene groove. The transducer can be placed on the supraclavicular fossa to visualize the BP first and is then relocated more medially. The T1 anterior ramus can be found separate from the undersurface of the BP (inferior cord) submerging under the first rib (Figure 5B and Supplementary Video S3).

### 3.4. How Can We Better Visualize Cervical Anterior Rami during the Scanning Process?

When we are trying to obtain a good quality US image, it is advised to position the target in the middle of the screen. This facilitates the examiner to be attentive to the primary focus as well as to appreciate better the whole regional anatomy. During the cervical anterior ramus imaging, it is important to know that the cervical spine has a lordotic curvature. Therefore, while sweeping the transducer from the cranial to the caudal aspect, movement should be gradual from posterior to anterior to locate the transverse processes of the cervical vertebrae in the center. However, if the transducer is merely moved vertically, certain anterior rami will fall at the edge of the scanning plane or might even become invisible.

### 3.5. Which Vascular Structure(s) Should We Be Aware Of?

**Figure 6:**
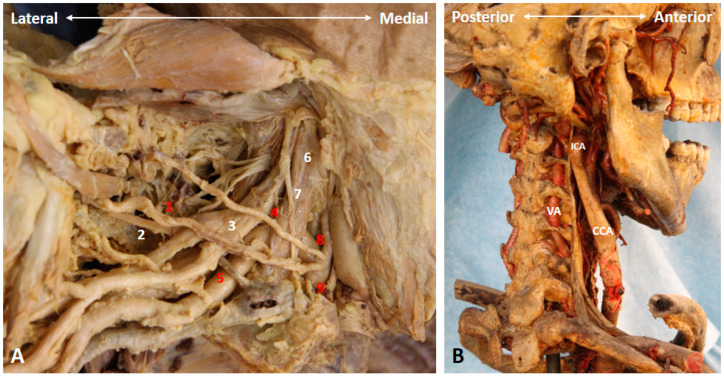
The transverse cervical artery, thyrocervical trunk, suprascapular artery and nerve (**A**), as well as the course and relevant vasculature of the vertebral artery (**B**) in a cadaver model. 1: suprascapular artery; 2: suprascapular nerve; 3: superior trunk; 4: transverse cervical artery; 5: subclavian artery; 6: anterior scalene muscle; 7: phrenic nerve; 8: inferior thyroid artery; 9: thyrocervical trunk. CCA: common carotid artery; ICA: internal carotid artery; VA: vertebral artery. Red numbers indicate arteries, while the white numbers indicate nerves and muscles.

**Figure 7:**
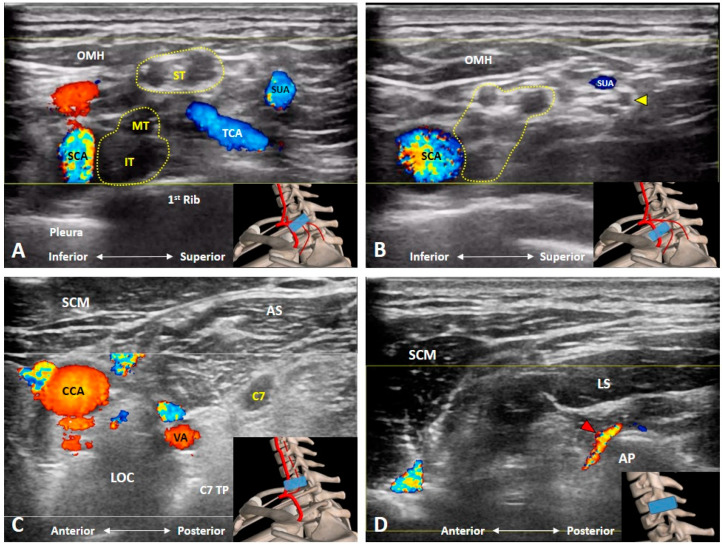
Doppler ultrasound imaging of the transverse cervical artery, thyrocervical trunk (**A**), suprascapular artery and nerve (**B**), vertebral artery (**C**), and radicular artery (**D**). AP: articular pillar; AS: anterior scalene muscle; CCA: common carotid artery; IT: inferior trunk; LOC: longus colli muscle; LS: levator scapulae muscle; MT: middle trunk; OMH: omohyoid muscle; SCA: subclavian artery; SCM: sternocleidomastoid muscle; ST: superior trunk; SUA: suprascapular artery; TCA: transverse cervical artery; VA: vertebral artery. Yellow arrowhead: suprascapular nerve; Red arrowhead: radicular artery. The yellow dashed area encircles the brachial plexus, and the blue rectangles in the bottom-right corners represent the transducer position.

At the supraclavicular portion of the BP, two vessels are noteworthy [5]. Transverse cervical artery emerging from the thyrocervical trunk and traversing posteriorly on top of or through the BP (Figure 6A and Figure 7A and Supplementary Video S4). The other vessel is the suprascapular artery, which is also a branch of thyrocervical trunk and courses beside the suprascapular nerve (Figure 6A and Figure 7B and Supplementary Video S4). Furthermore, the origin of the transverse cervical artery is closer to the root of the thyrocervical trunk than that of the suprascapular artery. Therefore, it is mostly seen coursing through the middle of the supraclavicular BP, whereas the suprascapular artery is usually visualized coursing above the supraclavicular BP.

Likewise, at the cervical root level, two vessels require extra attention [7]. The vertebral artery (branch of the subclavian artery) usually enters the transverse foramen over the C6 transverse process. At the level of C7, it is just anterior to the C7 anterior ramus (Figure 6B and Figure 7C). Of note, both structures may look similar without the use of Doppler US imaging. Additionally, during US-guided cervical anterior ramus injection, the needle is suggested to be introduced from posterior to anterior for preventing injury to the vertebral artery [6]. The second vessel to be precisely recognized is the radicular artery, which might be arising from the ascending and deep cervical arteries and entering the intervertebral foramen from the articular pillars [11]. Since it is mostly located posterior to the corresponding cervical anterior ramus, the examiner should be cautious if a posterior-to-anterior approach is used while they perform the cervical root injection (Figure 7D).

### 3.6. Which Branches of the BP Can Be Seen in the Cervical Region?

**Figure 8:**
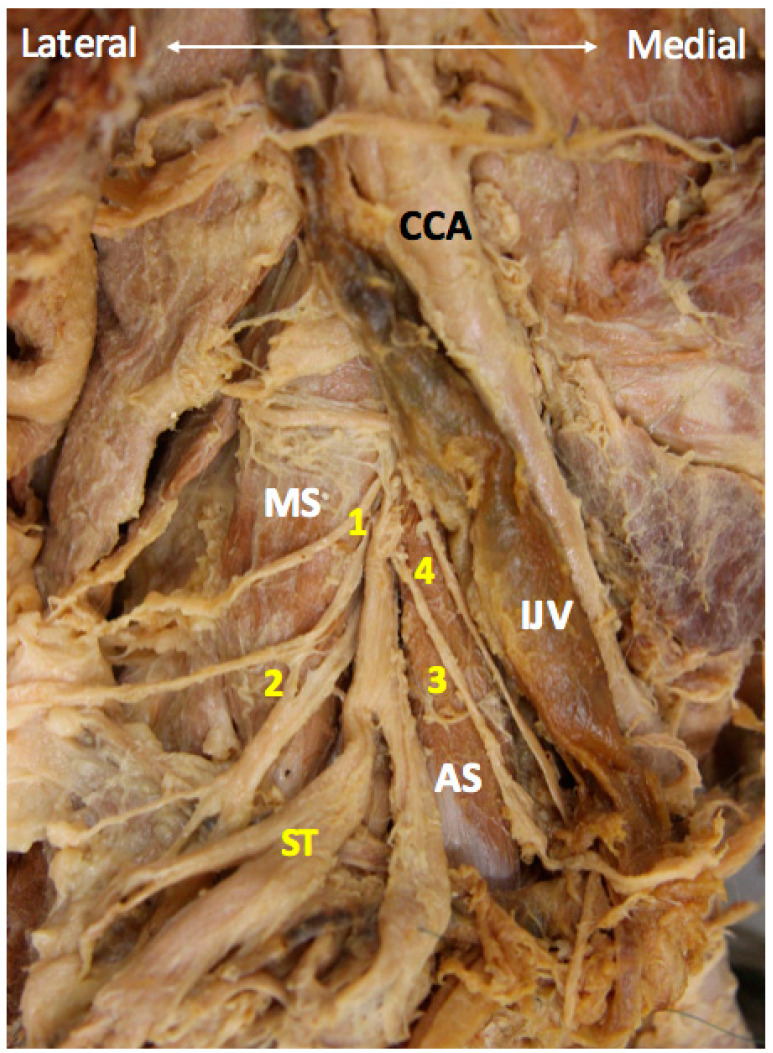
The long thoracic and dorsal scapular nerves cadaveric models. 1: dorsal scapular nerve; 2: long thoracic nerve; 3 phrenic nerve; 4: vagus nerve. AS: anterior scalene muscle; CCA: common carotid artery; IJV: internal jugular vein; MS: middle scalene muscle; ST: superior trunk.

**Figure 9:**
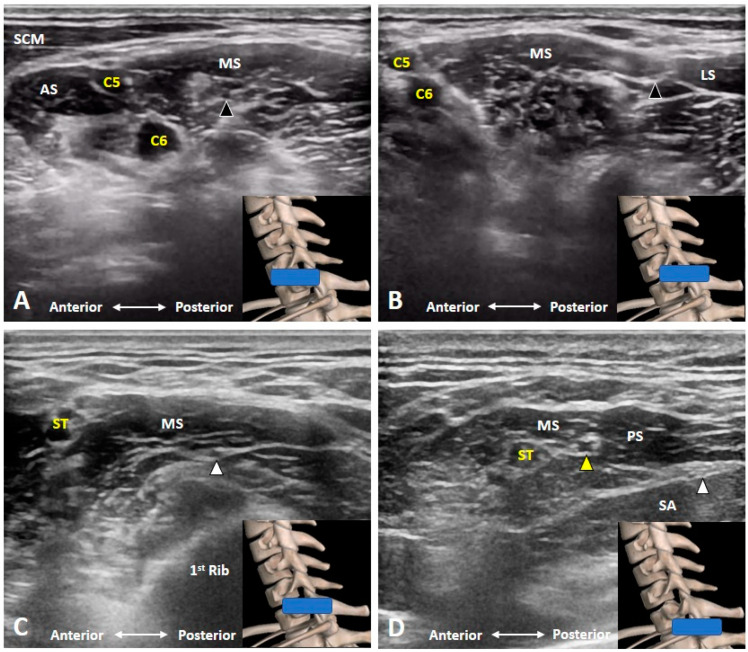
The dorsal scapular nerve (black arrowhead) emerges from the C5 anterior ramus, pierces the medial scalene muscle (**A**) and further courses underneath the levator scapulae muscle (**B**). The long thoracic nerve (white arrowhead) also travels inside the middle scalene muscle (**C**). The suprascapular nerve (yellow arrowhead) branches from the superior trunk of the brachial plexus (**D**). AS: anterior scalene muscle; LS: levator scapulae muscle; MS: middle scalene muscle; PS: posterior scalene muscle; SCM: sternocleidomastoid muscle; ST: superior trunk; SA: serratus anterior muscle. The blue rectangles at the bottom-right corners represent the transducer position.

Three branches of the BP can be visualized at this level. First of which is the dorsal scapular nerve emerging from the posterior aspect of the C5 anterior ramus [7]. As it pierces the medial scalene muscle (Figure 8 and Figure 9A) and further courses underneath the levator scapular muscle (Figure 9B and Supplementary Video S5), the transducer is suggested to be placed along the transection of the middle scalene muscle to retrieve better the short-axis view of the nerve. In certain variants, the dorsal scapular nerve pierces the anterior or posterior scalene muscle [12]. This nerve should be cautiously located and avoided when performing an US-guided C5 anterior ramus injection.

The second branch is the long thoracic nerve receiving fibers from the C5, C6 and C7 anterior rami [13]. Like the dorsal scapular nerve, it also traverses within the middle scalene muscle in most cases. However, it may pierce the anterior scalene or posterior scalene muscle in some variants [12]. As such, the transducer can be positioned along a similar but slightly inferior scanning plane as in the dorsal scapular nerve. To distinguish the two nerves, the transducer is needed to be swapped back and forth to ensure that one rootlet of the long thoracic nerve is derived from C6 or C7 anterior rami (Figure 9C and Supplementary Video S6). The nerve should be avoided during an US-guided interscalene block.

The third branch is the suprascapular nerve from the superior trunk of the BP [14]. Scanning can start from the C5 anterior ramus and gradually moving the transducer laterally until the C5 and C6 anterior rami merge to become the superior trunk. The suprascapular nerve usually emerges from the posterior aspect of the superior trunk (Figure 9D), whereas the anterior and posterior divisions are branched off from its anterior aspect.

## 4. Imaging for BP Pathologies

### 4.1. Radiation-Induced Brachial Plexopathy

Brachial plexopathy may develop after months to years after radiation therapy [15]. It is more likely to occur in patients radiated for breast, lung, head, and neck tumors. The causative mechanism can be direct radiation damage to vascular endothelium near the BP or secondary to progressive fibrosis of the skin, subcutaneous tissue and muscles over the neck [16]. The initial symptom can be paresthesia over the ipsilateral upper limb, which is usually ignored. Most of the patients seek help when they have weakness, intolerable pain, or muscle atrophy. The upper and middle trunks are commonly affected, leading to neurological complaints over the shoulder girdles and arms. In patients with breast cancer who are receiving irradiation to the axilla, the inferior trunk may be affected which causes symptoms over the hands and medial aspect of the upper limbs.

**Figure 10:**
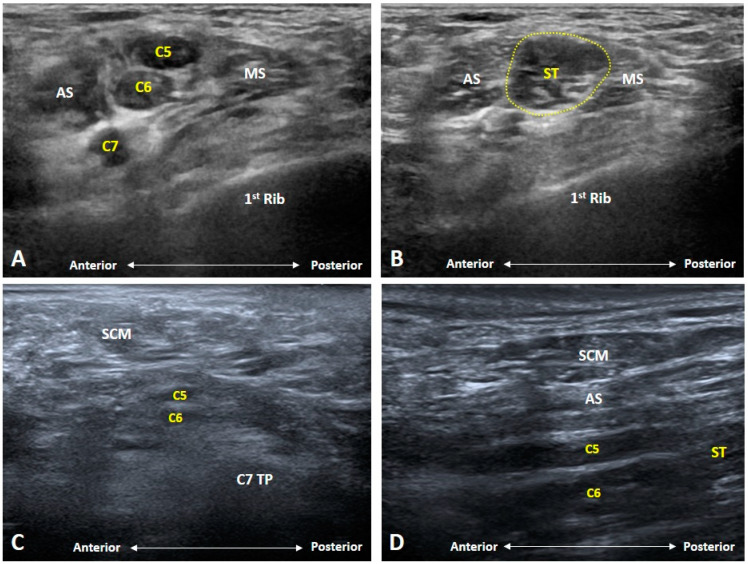
Ultrasound imaging of radiation-induced brachial plexopathy. Nerve fascicles of the brachial plexus at the root (**A**) and trunk (yellow dashed area) (**B**) levels appear enlarged with thickened epineurium. The muscles surrounding the neck are atrophic and fibrotic in the short-axis (**C**) and long-axis (**D**) views. AS: anterior scalene muscle; MS: middle scalene muscle; SCM: sternocleidomastoid muscle; ST: superior trunk.

There are several sonographic findings of radiation-induced brachial plexopathy [2]. First, the subcutaneous tissue over the supraclavicular fossa becomes extremely thin. Second, the muscles surrounding the neck become atrophic/fibrotic and involvement of the scalene muscles is usually associated with nerve compressive symptoms. Third, the nerve fascicles of the upper and middle trunks appear enlarged with thickened epineurium (Figure 10). While the size of the BP at the root level is relatively normal, the nerve tracks adhere to each other with minimal reciprocal movements of the upper limbs and neck. In patients with an early diagnosis of brachial plexopathy due to compression of the fibrotic tissues, hydro-dissection under US guidance may be helpful for symptom relief or decelerating symptom progression (Supplementary Video S7).

### 4.2. Neoplastic/Metastatic Brachial Plexopathy

Brachial plexopathy can be caused by primary and metastatic tumors [17]. The former only accounts for less than 5% of upper extremity malignancies and usually has a neurogenic origin e.g., schwannoma, neurofibroma, and malignant nerve sheath tumor [18]. The latter is primarily from malignancies of the lung, breast, thyroid, and bone through direct invasion and lymphatic/hematologic spread. In case of Pancoast tumor, a neoplasm of the lung apex, the involvement of the BP is common. Unlike radiation-induced BP lesions, malignancy-related brachial plexopathy is likely to affect the lower trunk, probably due to its proximity to the location of the primary tumors. Initial symptom is pain over the shoulder girdle and upper limb. Muscle weakness is most likely to develop in the later stages.

**Figure 11:**
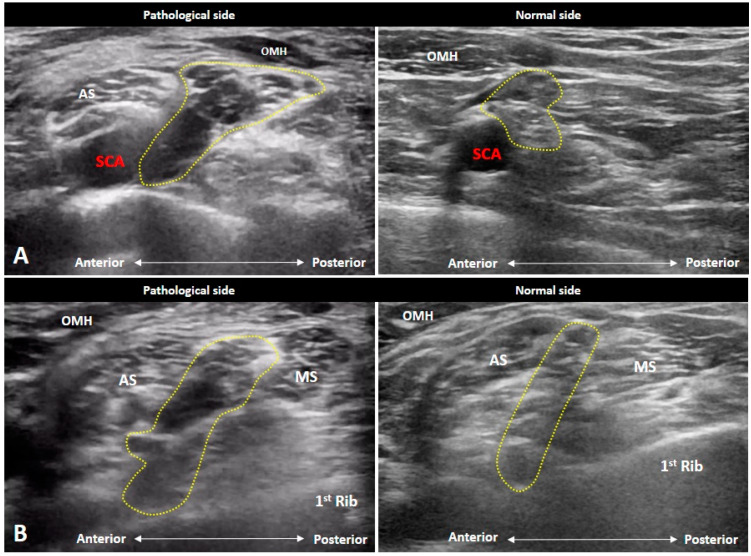
Ultrasound imaging of metastatic brachial plexopathy. The nerve fascicles (yellow dashed area) at the trunk level (**A**) and intertubercular groove (**B**) are enlarged secondary to the tumor infiltration. Like elsewhere, side-to-side comparison is contributory. AS: anterior scalene muscle; MS: middle scalene muscle; OMH: omohyoid muscle; SCA: subclavian artery.

**Figure 12:**
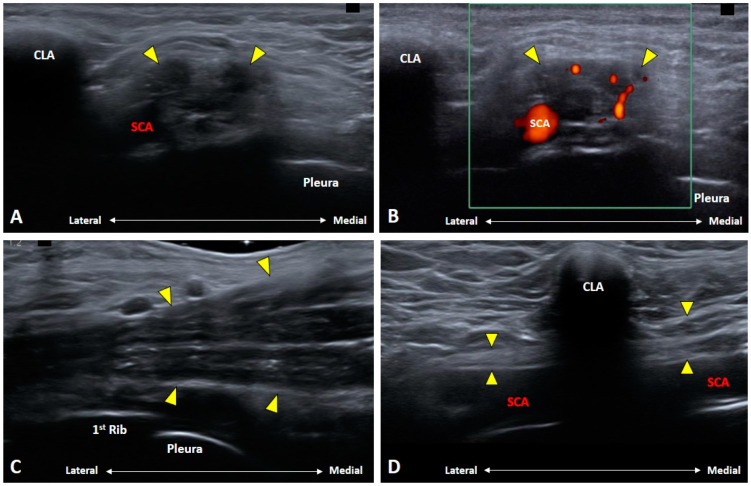
Ultrasound imaging of metastatic brachial plexopathy. The nerves (yellow arrowheads) are enlarged (**A**) with intra-neural hypervascularity (**B**). In the long-axis view, segmental swelling of the nerves (yellow arrowheads) are seen at the supraclavicular (**C**) and infraclavicular (**D**) levels. CLA: clavicle; SCA: subclavian artery.

US imaging usually shows enlargement of the affected nerves secondary to tumor infiltration (Figure 11 and Figure 12A) [17]. The transducer can be pivoted to align with the long axis of the nerves, which may show segmental fusiform swelling (Figure 12C,D). Perineural hypervascularity is also common in the Doppler mode. The lesion is more likely to be metastatic if an increased degree of hyperemia is found (Figure 12B) although intralesional vascularity can be seen in some primary tumors such as peripheral sheath tumors. Unlike radiation-induced brachial plexopathy, the epineurium is not thickened, and the echotexture of the surrounding muscles is mostly normal.

### 4.3. Parsonage-Turner Syndrome (Neuralgic Amyotrophy)

Parsonage-Turner Syndrome, also known as neuralgic amyotrophy or idiopathic brachial neuritis, is manifested by sudden onset of pain over the shoulder girdle and upper limb [19]. Bilateral involvement is relatively rare. Neurological symptoms usually develop after an infection, recent immunization, and rheumatic diseases e.g., systemic lupus erythematosus, temporal arteritis, and polyarteritis nodosa. Besides severe pain, patients also suffer weakness over the involved muscles. The sensory deficit is relatively uncommon compared with motor impairment.

**Figure 13:**
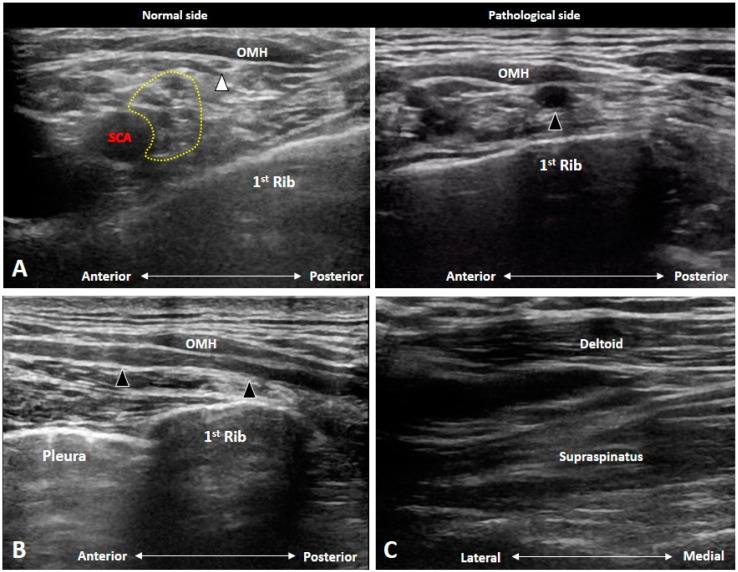
Ultrasound imaging of the suprascapular nerve (black arrowheads) in a patient with Parsonage-Turner syndrome (**A**). The normal nerve (white arrowhead) on the contralateral side (**A**). The enlarged suprascapular nerve in the long-axis view (**B**), atrophy and fat infiltration of the supraspinatus and infraspinatus muscles (**C**) are also seen. OMH: omohyoid muscle; SCA: subclavian artery. The yellow dashed area encircles the brachial plexus.

As shoulder pain serves as the predominant symptom of Parsonage-Turner Syndrome, US imaging is needed to scrutinize possible shoulder disorders. The only pathology that can cause the same severity of pain as Parsonage-Turner Syndrome would perhaps be calcific tendinopathy in the resorptive phase. If the investigator finds that the severity of the rotator cuff lesion is not proportional to the pain, Parsonage-Turner Syndrome should be highly suspected. Tracking of the suprascapular nerve may be helpful in the diagnosis as the majority of the patients have involvement of this nerve (Figure 13A,B). According to the study conducted by Gruber et al. [20], the average cross-sectional area of the suprascapular nerve at the mid-clavicular level was 6.36 mm^2^ in patients and 2.79 mm^2^ in controls. A cut-off value of 4.2 mm^2^ was shown to have the best diagnostic performance. Furthermore, atrophy and fat infiltration of the supraspinatus and infraspinatus muscles are also common at the late stage (Figure 13C). However, the sonographer still needs to scrutinize other causes that can lead to suprascapular nerve entrapment, such as a paralabral cyst.

### 4.4. Brachial Plexus Trauma

The BP can be traumatized through three main mechanisms: contusion, overstretching and penetration [21]. The best imaging modality for assessing brachial plexus trauma is perhaps MRI [22], which is capable of evaluating the preganglionic and postganglionic lesions. Whereas US imaging is mostly limited to the assessment of postganglionic abnormalities. There is only one type of preganglionic lesion than can be depicted by US imaging i.e., pseudomenigocele [1]. It ensues with abnormal accumulation of the cerebrospinal fluid due to a tear in the dura. US imaging might reveal a lobulated fluid collection emerging from the intertubercular groove with visualization of normal anterior rami.

**Figure 14:**
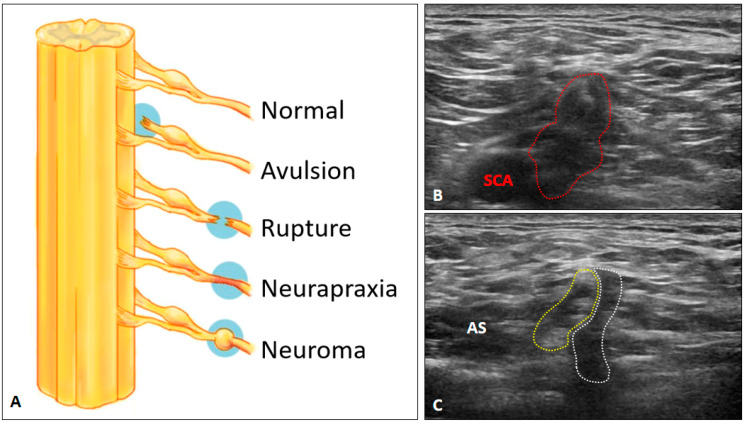
Schematic drawing of different types of nerve injury (**A**). Ultrasound imaging of chronic traumatic brachial plexus injury: a neuroma (red dashed area) (**B**) and tethered brachial plexus (yellow dashed area) besides the scar (white dashed area) (**C**). AS: anterior scalene muscle; SCA, subclavian artery.

Seddon’s classification [23] has been widely used in the evaluation of peripheral nerve injuries including the postganglionic BP lesions. Neurapraxia is the mildest type with the intact endoneurium, perineurium, and epineurium. The axonal damage is minimal and only conduction block would be observed in electrophysiological studies. US imaging may reveal nearly normal nerve tracts or mildly swollen nerve fascicles. Axonotmesis indicates disruption of axonal continuity but with the preservation of the surrounding connective tissues. US imaging would show significant swelling of the damaged nerves despite the epineurium appearing grossly intact. Neurotmesis is the worst type wherein total disruption of the injured nerves is seen (Figure 14A). US imaging reveals a visible gap of the nerve tract and retraction of the completely transected ends. In chronic cases, a neuroma may also develop (Figure 14B) and a hyperechoic scar can be seen along the injured nerves (Figure 14C).

### 4.5. Thoracic Outlet Syndrome

Thoracic outlet syndrome (TOS) pertains to compression of the neurovascular structures in the cervico-axillary region dynamically [24]. It can be provoked by the posture e.g., side bending/rotation of the neck, excessive abduction of the arm and hyper-retraction of the scapulae. The causes of the syndrome can be neurogenic or vascular (subclavian artery or vein) compression [25] whereby several physical tests (e.g., Adson, costoclavicular, Roos and Wright) have been used in the diagnosis, mainly to examine vascular compression [24]. The three regions where the BP can be compressed are the interscalene triangle, costoclavicular space and the retro-pectoralis minor region.

**Figure 15:**
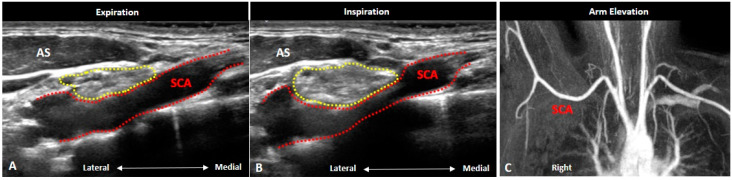
Ultrasound imaging of thoracic outlet syndrome caused by subclavian artery compression in a young woman suffering right arm pain during inspiration. Ultrasound images during expiration (**A**) and inspiration (**B**) showed sudden narrowing of the subclavian artery during inspiration (red dashed area). Focal stenosis of the subclavian artery was not obvious in the magnetic resonance angiography during right arm elevation (**C**). Inferior trunk: yellow dashed area. AS: anterior scalene muscle; SCA: subclavian artery.

US scanning should be performed continuously from the interscalene triangle until the retro-pectoralis minor region. Dynamic examination during any of the aforementioned tests may be helpful to provoke the symptoms. The transducer can be pivoted to align with the long axis of the BP to check any focal nerve encroachment. The patency of the subclavian artery and vein should be scrutinized as well. Color Doppler mode with spectral analysis can also be employed to detect hemodynamic changes of the subclavian vessels during provocative postures (Figure 15). Individuals with variations of the brachial plexus piercing the scalene muscles may be associated with neurogenic TOS [26]. In certain cases of neurogenic TOS, the lower trunk of the brachial plexus may be indented by the fibromuscular bands, leading to a wedge-sickle sign on US imaging [27]. Finally, it should be kept in mind that the costoclavicular space is a potential blind spot for US imaging. If needed, MRI should be prescribed to rule out any hidden lesions.

## 5. Conclusions

US imaging allows the clear depiction of the BP and is capable of being the first line to image relevant disorders. Familiarization with regional bony and muscular anatomy is definitely prerequisite before one can use US in the diagnosis and guided interventions of various BP pathologies. Lastly, physicians must be aware of the limitations of US imaging and should not hesitate to prescribe MRI in case of a suspicious preganglionic lesion.

## Supplementary Materials

The following are available online at https://www.mdpi.com/2075-4418/10/5/324/s1.
